# Molecular detection and identification of *Wolbachia* in three species of the genus *Lutzomyia* on the Colombian Caribbean coast

**DOI:** 10.1186/s13071-017-2031-x

**Published:** 2017-02-28

**Authors:** Rafael José Vivero, Gloria Cadavid-Restrepo, Claudia Ximena Moreno Herrera, Sandra I. Uribe Soto

**Affiliations:** 1 Universidad Nacional de Colombia at Medellín, Medellín, Colombia; 2 Grupo de Investigación en Sistematica Molecular, Universidad Nacional de Colombia at Medellín, Medellín, Colombia; 3 PECET (Programa de Estudio y Control de Enfermedades Tropicales), Universidad de Antioquia, Medellín, Colombia; 4 Grupo de Microbiodiversidad y Bioprospección, Laboratorio de Biología Celular y Molecular, Universidad Nacional de Colombia at Medellín, Medellín, Colombia

**Keywords:** *Wolbachia*, Phylogroup wLeva, *wsp* gene, *Lutzomyia*, Natural infection

## Abstract

**Background:**

The hematophagous habits of insects belonging to the genus *Lutzomyia* (Diptera: Psychodidae), as well as their role as biological vectors of *Leishmania* species, make their presence an indication of infection risk. In the present study, seven species of *Lutzomyia* were identified and screened for natural infections with *Wolbachia.*

**Methods:**

Collection of sand flies was done in an endemic focus of leishmaniasis on the Colombian Caribbean coast (Department of Sucre, Ovejas municipality). DNA collected from *Lutzomyia* species was evaluated with PCR for *wsp* gene amplification to screen for bacterial infection.

**Results:**

Endosymbiotic *Wolbachia* was found in three species: *Lutzomyia c. cayennensis, Lutzomyia dubitans* and *Lutzomyia evansi*. Two *Wolbachia* strains (genotypes) were found in *Lutzomyia* spp. These genotypes were previously unknown in dipteran insects. The *wLev* strain was found in *Lutzomyia dubitans*, *L. c. cayennensis* and *L. evansi* and the *wLcy* strain was found only in *L. c. cayennensis.*

**Conclusions:**

Genetic analysis indicated that the *Wolbachia* strains *wLcy* and *wLev* belong to the B Supergroup. This study provides evidence of infections of more than one strain of *Wolbachia* in *L. c. cayennensis.*

**Electronic supplementary material:**

The online version of this article (doi:10.1186/s13071-017-2031-x) contains supplementary material, which is available to authorized users.

## Background

*Los Montes De María* is a region located on the Caribbean coast of Colombia which has been historically considered as a focus of several clinical forms of leishmaniasis [1]. In this region, the municipality of Ovejas (Department of Sucre) is of particular epidemiological interest due to the endemic character of leishmaniasis that is occurring in urban, peri-urban and rural areas there. The diversity of *Lutzomyia* spp. (vector insects) present in Ovejas is high and most of the species are implicated in leishmaniasis transmission [2, 3].

In Latin America, vector control campaigns developed for leishmaniasis have mainly focused on chemical control using synthetic pesticides such as pyrethroids and chlorofluazuron [4]. The use of biological alternatives or their derivatives (bacteria, sex pheromones, entomopathogenic fungi and toxic plants) have also been considered, but few are used by vector control agencies in Colombia [2]. The medical importance of phlebotomine sand flies (particularly those of the *Lutzomyia* species) points to the need to consider new and more effective control measures, including some that have already been used for the control of other insects transmitting vector-borne diseases. Among such methods is transfection with bacteria of the genus *Wolbachia* [5].

Bacteria in the genus *Wolbachia* are intracellular microorganisms belonging to α-proteobacteria (Rickettsia), have maternal inheritance and are commonly found in insect intestines, salivary glands, ovaries and thoraces [6, 7]. These bacteria may affect the reproductive capabilities of their hosts through diverse mechanisms, generating effects such as the death of male offspring as well as feminization and cytoplasmic incompatibility (CI) [8]. The pathogenic effect of some phenotypes of *Wolbachia* is now being evaluated on viruses such as Zika, dengue and chikungunya, as well as on *Plasmodium* [9, 10].

The use of certain strains of *Wolbachia* is considered to be a promising alternative for decreasing the population density of *Lutzomyia* species and interfering with the multiplication of parasites and, as a result, *Leishmania* transmission [11–13]. Thus, initial research efforts have been directed toward screening the presence and circulation of *Wolbachia* strains in these and other vectors [14, 15].

In the Americas, only five species of the genus *Lutzomyia* have been found to have low levels of *Wolbachia* infection, with strains belonging to the A and B Supergroups: *Lu. cruciata* in México, *Lu. trapidoi* and *Lu. vespertilionis* in Panamá and *Lu. whitmani* in Brazil. In Colombia, only *Lu. shannoni* was reported as positive for *Wolbachia* presence [16–18]. Supergroup A, also includes the *Wolbachia* species detected in *Sergentomyia* and *Phlebotomus* [19–21]. Currently, genes (16S *rRNA*, *ftsZ*, *wsp* gene) and techniques (Multilocus Sequence Typing technique MLST) are being used to validate the identification and phylogeny of strains of *Wolbachia* [22].

Partial *wsp* gene sequences exhibited informative characters useful in the identification of *Wolbachia* strains detected in *Lutzomyia* spp. The *wsp* gene has evolved at a much faster rate than any previously reported gene in *Wolbachia* [19–22]. Due to this reason, its nucleotide variability facilitates the division into Subgroups and Groups in a consistent manner [22]. The nucleotide variability of the *wsp* gene and the combination of different primers in PCR reactions is an approach that enables a fast assigning of unknown strains to a particular group, due to its specificity and lack of cross-reactions.

The aim of the present study was molecular detection and identification of the endosymbiont *Wolbachia* in natural populations of *Lutzomyia* species found in the municipality of Ovejas on the Colombian Caribbean coast, as well as an analysis of the gene sequence coding for the main surface protein of endosymbiotic *Wolbachia* (*wsp*).

## Methods

### Phlebotomine survey, processing and identification

Sand flies were collected in peri-urban environments in the municipality of Ovejas (75°13'E; 9°31'N; 277 m above sea level) during an entomological survey performed between February 21 and 27, 2013. This location is classified as a tropical dry forest ecosystem. Collection was done using CDC white light traps, located indoors and near homes, overnight, between 17:00 and 06:00 h. Shannon traps were also used for collection near homes. Additionally, diurnal collection using a mouth aspirator was done in the vicinity of nocturnal trapping sites. Collected specimens were kept dry in 1.5 ml vials and transported to the laboratory with dry ice. Once at the laboratory, they were kept at −20 °C. The head and last three abdominal segments were removed from the specimens in order to perform taxonomic identification following the Young & Duncan classification system [23]. The thorax and remaining abdominal segments were stored at −20 °C until DNA extraction.

### Pool formation and DNA extraction

Following taxonomic identification, males and females were separated by species in groups with a variable number of individuals (6 to 10) in 1.5 ml Eppendorf tubes. The formation of groups in this way is justified by differences in the abundance of species in the study area, which complicates statistical interpretation regarding *Wolbachia* infection rates, but increases the success of molecular detection of bacteria found in natural populations of *Lutzomyia* in the conditions encountered. In addition, the samples were all collected at the same time.

**Fig. 1:**
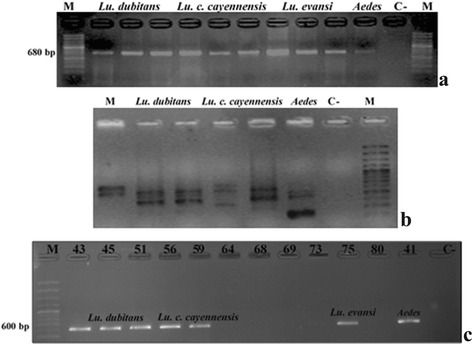
PCR from *Lutzomyia* genomic DNA pools. **a** PCR amplification of the *cox*1 gene fragment to evaluate the quality of DNA and absence of inhibitors from genomic DNA pools. **b** PCR for ITS to estimate the quality of available bacterial DNA. **c** PCR amplification from a partial fragment of *wsp* gene with primers *wsp* in different species of *Lutzomyia.* PCR products were evaluated in 1% electrophoresis gels. *Abbreviations*: M, a 100 bp DNA ladder; C-, negative control

DNA extraction was done according to the high salt concentration protocol [24]. The quality of DNA (260/A280 ratio) and concentrations was analysed by Spectrophotometry (Thermo Scientific™ NanoDrop, Wilmington, USA). Additionally, a partial fragment of the cytochrome *c* oxidase subunit 1 (*cox*1) gene was amplified (Fig. 1) and the spacer region (ITS) between the 23S and 16S ribosomal gene (Fig. 1), in order to evaluate the quality of DNA present, as well as the absence of PCR inhibitors.

### PCR, cloning and DNA fragment sequencing for *Wolbachia wsp* gene

Primers *wsp*81F (5'-TGG TCC AAT AAG TGA TGA AGA AAC-3') and *wsp*691R (5'-AAA AAT TAA ACG CTA CTC CA-3') were used to amplify a partial fragment (600 bp) of the gene coding for the main surface protein of endosymbiotic *Wolbachia* (*wsp*) (Fig. 1) [25]. The reaction mix used to detect *Wolbachia* included 80 ng of sample DNA according to the conditions previously described [26, 27]. High fidelity Taq DNA Polymerase (Thermo Scientific, Wilmington, USA) was employed, as well as a conventional thermocycler (BIOMETRA). As a PCR positive control, DNA from ten *Aedes* (*Stegomyia*) *aegypti* larvae (kindly donated by the insectary of the PECET group) infected under laboratory conditions with a reference strain of *Wolbachia* (Supergroup A, strain *wMel*) were included (Fig. 1). As a PCR negative control, ultrapure water and DNA of *Aedes* (*= Stegomyia*) *aegypti* without *Wolbachia* was included (Fig. 1)*.*

*Wsp* gene amplicons were ligated into JET1.2 vectors (Thermo Scientific) and then transformed into *DH5α Escherichia coli*. At least five independent clones were sequenced for each positive sample involved in detecting *Wolbachia* strains to generate consensus sequences for further analysis, as well as to mitigate the potential of a mixed infection in the pools [27]. Clones with the partial products of *wsp* were verified by sequencing in both directions using universal primers from Macrogen Inc., Korea. For each assay, a negative control (no DNA) as well as a positive control (control PCR product by the cloning kit) was included.

### Identity of *Wolbachia* strains and their positions in phylogroups

The *wsp* gene obtained from *Wolbachia* were sent for sequencing (Macrogen, Korea) and the results were compared to previously identified sequences using the basic local alignment search tool (BLASTN) (https://www.ncbi.nlm.nih.gov/) and edited with Bioedit v.7.2.5 [28] in order to obtain detected consensus sequence for every *Lutzomyia* species. This was also made with gene sequences of *Wolbachia*, which were available in the National Center for Biotechnology Information (NCBI) database and *Wolbachia* MLST database (http://pubmlst.org/wolbachia/). The nucleotide alignment reading framework reported by O'Neill (ftp://ftp.ebi.ac.uk/pub/databases/embl/align/; Access Number DS42468) was considered, which suggests starting the analysis by translating the sequences to amino acids as a guide to align the DNA sequences of the *wsp* gene [27].

Alignments of sequences of *wsp* genes obtained in *Lutzomyia* and reported in GenBank (Additional file 1) were performed using the Clustal W and Muscle algorithms incorporated in MEGA 6. Verification of recombination events and the presence of chimeras was performed with RDP4 (Recombination Detection Program version 4) software, using all sequences of *wsp* obtained in this study in order to ensure the accuracy of nucleotide variability with respect to previously reported sequences in GenBank (Additional file 1). Patterns of genetic divergence (nucleotide composition, number of haplotypes, variable sites) and K2P genetic distances were evaluated using Bioedit v.7.2.5 and DNAsp 5.0 software.

All aligned sequences (= haplotypes) of *wsp* genes obtained in this study and reported in GenBank were exported using MEGA software. Description codes include the following abbreviations for species: *Lev*, *Lutzomyia evansi*; *Lcy*, *Lutzomyia c. cayennensis* and *Luduv*, *Lutzomyia dubitans* followed by the letters *ov,* which refer to the place where they were collected in Colombia (ov, municipality of Ovejas) and numbers corresponding to specimens with the same sequence.

Subsequently, the identities and relationships of the *Wolbachia* strains obtained in our study was determined by performing a phylogenetic inference analysis using the Bayesian method (number of generations = 1,000,000) with the MrBayes 3.0 software under the substitution model GTR + G (number of estimated parameters k = 139; Akaike information criterion (AIC) = 7807.8819); with jModeltest 2.1.4 software [29]; and Phyml 3.0 software [30]. All of the sequences obtained in the present study (KR907869–KR907874) were submitted to GenBank (Additional file 1).

### PCR amplification of the HSP-70 N *Leishmania* gene in female groups

A PCR test was done to screen *Leishmania* infection in females of *Lutzomyia*. The primers used were HSP70-F25 (5'-GGA CGC CGG CAC GAT TKC T-3') and HSP70-R617 (5'-CGA AGA AGT CCG ATA CGA GGG A-3'), which amplify a 593 bp partial segment of the *HSP-70 N* gene (coding for Heat shock protein 70) [31]. PCR testing was done following the conditions and thermal profile described by Fraga et al. [31]. As a positive control, DNA from *Leishmania panamensis* (reference strain UA140) and *Leishmania braziliensis* (reference strain UA 2903), which was kindly provided by the PECET group of the Universidad de Antioquia, was included.

## Results

### Taxonomic identification of sand flies

A total of 325 individuals were collected from peri-urban environments. Morphological and taxonomic guides allowed the identification of seven species: *Lu. evansi*, *Lu. trinidadensis*, *Lu. c. cayennensis*, *Lu. dubitans*, *Lu. gomezi*, *Lu. rangeliana* and *Lu. atroclavata* (Table 1). *Lutzomyia dubitans* (110 specimens; 33.8%) and *Lu. c. cayennensis* (107 specimens; 32.2%) were the species found in the highest proportions (Table 1). Thirty-five pools were formed according to sex and taxonomic assignation as described above.

**Table 1:** Formation of pools of *Lutzomyia* spp. for detection of infection by *Wolbachia* in the peri-urban environments in the municipality of Ovejas, Department of Sucre, Colombia

Species	Sex	No. of pools formed (No. of specimens)	No of positive pools with *Wolbachia* (%)	Total no. of specimens per species analysed (%)
*Lu. dubitans*	Female	8 (80)	2 (5.7)	110 (33.8)
	Male	3 (30)	1 (2.8)	
*Lu. c. cayennensis*	Female	4 (40)	1 (2.8)	107 (32.2)
	Male	7 (67)	2 (5.7)	
*Lu. gomezi*	Female	3 (24)	0	41 (9.5)
	Male	2 (17)	0	
*Lu. trinidadensis*	Female	1 (7)	0	33 (10.1)
	Male	3 (26)	0	1
*Lu. rangeliana*	Female	1 (10)	0	16 (4.9)
	Male	1 (6)	0	
*Lu. evansi*	Male	1 (8)	1 (2.8)	8 (2.4)
*Lu. atroclavata*	Female	1 (10)	0	10 (3)
Total	–	35 (325)	7 (20)	325 (100)

### *Wolbachia* (*wsp* gene) infection

As expected, all PCR fragments of the *wsp* gene were approximately 600 bp in size, and were obtained from three species: *Lu. dubitans*, *Lu. c. cayennensis* and *Lu. evansi.* Among these three sand fly species, seven pools were positive for *Wolbachia* (Fig. 1, Table 1). Low relative infection rates were found in *Lu. dubitans* and *Lu. c. cayennensis* (3 positive pools; 8.5% for both species) (Table 1). In *Lu. evansi* (1 positive pool; 2.8%), only one group was positive. It worth noting that *Wolbachia* was present in both sexes of *Lutzomyia*, particularly in *Lu. dubitans* (males, 5.7%; females, 2.8%) and *Lu. c. cayennensis* (males, 5.7%; females, 2.8%) (Table 1), while in *Lu. evansi Wolbachia* was detected only in males. *Lutzomyia rangeliana*, *Lu. trinidadensis*, *Lu. gomezi* and *Lu. atroclavata* were all negative for *Wolbachia.* The positive control strain *wMel* successfully amplified in all PCR assays of the *wsp* gene for *Wolbachia* and the negative controls showed no PCR products.

### *Wolbachia* identity based on comparisons with previous sequences and assignation of phylogroups using *wsp gene* sequences

**Fig. 2:**
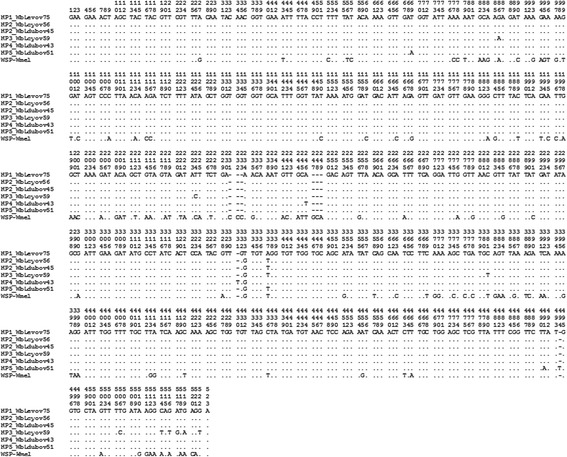
Multiple alignment of partial nucleotide sequences of *wsp* gene (Positions 1–523) of *Wolbachia* strains, detected in *Lutzomyia* species (*blue*) collected in Ovejas (Sucre, Colombia). Description codes include the following abbreviations for species: *Lev*, *Lutzomyia evansi*; *Lcy*, *Lutzomyia c. cayennensis*, and *Luduv*, *Lutzomyia dubitans* followed by *ov*, referring to the place of collection in Colombia (ov, municipality of Ovejas) and numbers corresponding to specimens with the same sequence. The haplotypes are: HP1, *WbLevov75*; HP2, *WbLcyov56/WbLdubov45*; HP3, *WbLcyov59*; HP4, *WbLdubov43*; and HP5, *WbLdubov51*. strain *wMel* is the positive control

**Fig. 3:**
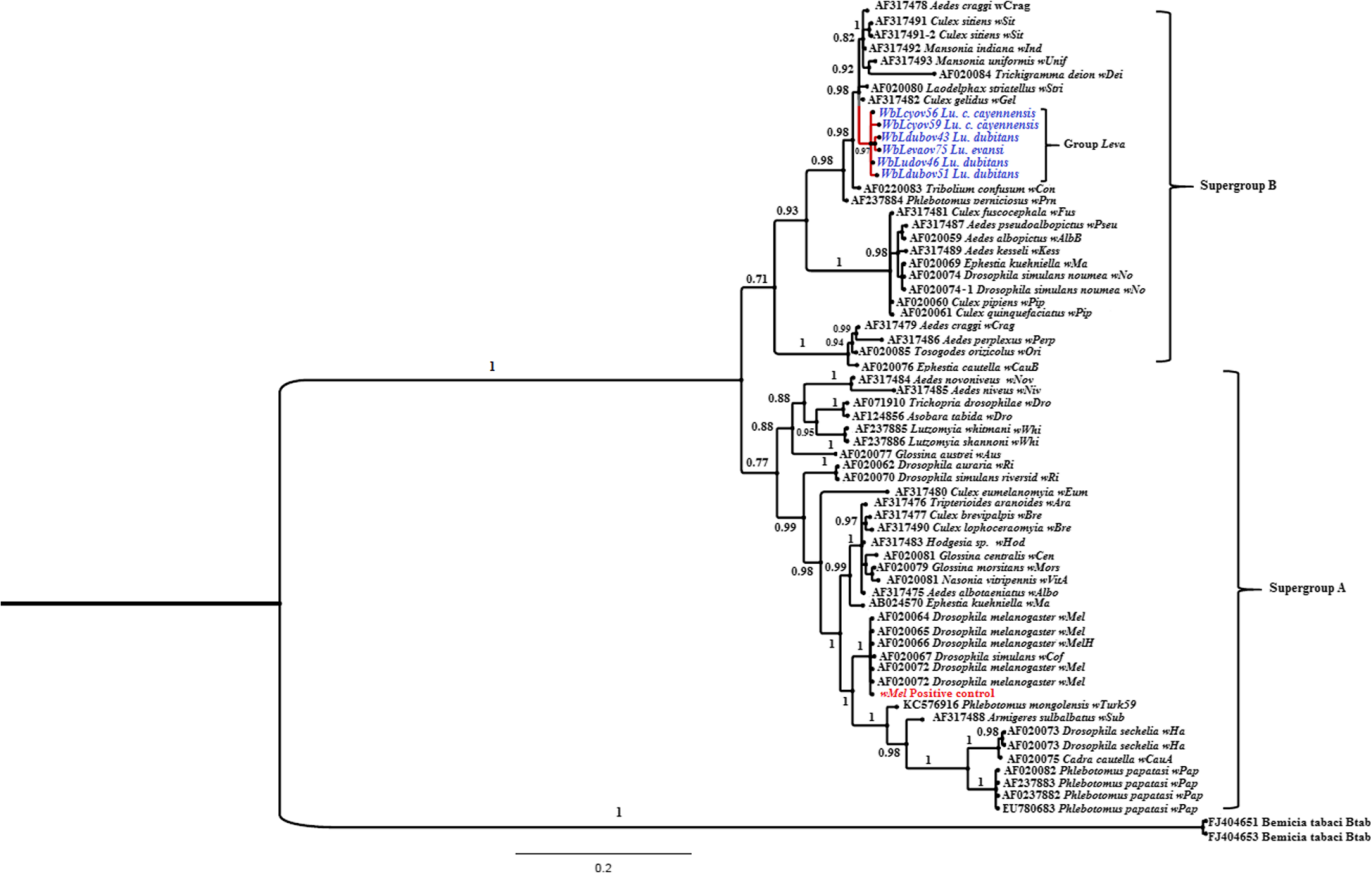
Phylogenetic relationships of *Wolbachia* strains inferred using *wsp* gene including the ones detected in *Lutzomyia* species (*blue*) collected in Ovejas (Sucre, Colombia). Numbers in nodes represent Bayesian posterior probabilities. Reconstruction performed with MrBayes (version 3.0). The *wMel* positive control is indicated in *red*

Based on DNA sequences, the presence and identity of *Wolbachia* in *Lu. dubitans*, *Lu. evansi* and *Lu. c. cayennensis* was determined. Nucleotide variability analysis based on fragments of 523 bp, showed only 15 variable sites among *wsp* sequences of *Wolbachia* obtained from *Lutzomyia* species (Fig. 2). In the Bayesian inference, 59 partial sequences of the *Wolbachia wsp* gene were included from strains related to arthropods, which are located in supergroups A and B, representing 24 groups with 57 previously detected strains from a wide number of insects (Additional file 1). Five haplotypes (HP) of the *wsp* gene (HP1 to HP5) were found in this study, which were described with short codes that allow the location of *Wolbachia* genotypes to be determined in relation to the species in which they were detected and that facilitate locating them in the tree created with all the sequences by Bayesian inference (Fig. 3).

The five haplotypes HP1 (*WbLevov75*); HP2 (*WbLcyov56/WbLdubov45*); HP3 (*WbLcyov59*); HP4 (*WbLdubov43*); and HP5 (*WbLdubov51*) differed due to 2–11 insertion-deletion and point mutation events (Fig. 2). The values of Kimura 2-parameter pairwise genetic distances among the haplotypes of *Wolbachia* were between 0.004 and 0.021 (Table 2), suggesting the existence of different strains. The haplotypes *WbLevov75*, *WbLcyov56*, *WbLdubov45*, *WbLdubov51* and *WbLdubov43*; representing the *wLev* strain, showed low levels of genetic differentiation (0.004) and high similarity (99.6%) (Table 2).

**Table 2:** Values of genetic distances K2P and percent of sequence identity based on alignment of the *wsp* gene among strains of *Wolbachia* in the Leva, Con, Unif and Pern groups (Supergroup B) and some strains (*WNiv* and *wWhi*) of Supergroup A

	Supergroup B									Supergroup A	
Groups	Leva		Unif		Con				Per	Niv	Whi
Strain	*wLev* ^(%)^	*wLcy* ^(%)^	*wUnif* ^(%)^	*wInd*	*wSit* ^(%)^	*wCon*	*wGel*	*wStri*	*wPer* ^(%)^	*wNiv* ^(%)^	*wWhi*
*wLev*	0.004^**99.6**^										
*wLcy*	0.017–0.021^**97.9**^	**–**									
*wUnif*	0.017–0.026^**97.4**^	0.019^**98.1**^	**–**								
*wInd*	0.014–0.019^**98.1**^	0.019^**98.1**^	0.010^**99.0**^	**–**							
*wSit*	0.020–0.026^**97.4**^	0.024^**97.6**^	0.014	0.014	**–**						
*wCon*	0.022–0.027^**97.3**^	0.027^**97.6**^	0.022^**97.8**^	0.012	0.017^**98.3**^	**–**					
*wGel*	0.014–0.019^**98.1**^	0.019^**98.1**^	0.014	0.014	0.010	0.012	**–**				
*wStri*	0.019–0.024^**97.6**^	0.019^**98.1**^	0.010	0.019	0.015	0.010	0.016	**–**			
*wPer*	0.014–0.020^**98**^	0.017^**98.3**^	0.017	0.027	0.022^**97.8**^	0.017	0.014	0.022	**–**		
*wNiv*	0.234– 0.255^**74.5**^	0.240^**76**^	0.229^**77.1**^	0.216	0.223^**77**^	0.226	0.222	0.219	0.219^**78.1**^	**–**	
*wWhi*	0.174–0.178^**86.6**^	0.167^**83.3**^	0.183^**81.7**^	0.176	0.173^**82.7**^	0.179	0.173	0.176	0.173^**82.7**^	0.136^**86.4**^	**–**

*Note*: The superscripts indicate the percent similarity between the sequences and were determined only among some strains representing different levels of variation: within the same strain, between strains of the same group, between strains of different groups, among strains from different supergroups

The haplotype *WbLcyov59-*HP3, representing the *wLcy* strain, exhibited similarity of 97.9% and higher values for genetic distances (0.017–0.021) when compared with haplotypes of the *wLev* strain (Table 2)*.* The *wLev* strain was present in *Lu. c. cayennensis*, *Lu. dubitans* and *Lu. evansi*, but in *Lu. c. cayennensis* the *wLcy* strain was also detected.

The *wLev* strain (Table 2), showed low levels of genetic differentiation as compared to the strains from Supergroup B, as well as showing affinity for strains from the groups Unif (*wUnif* = 0.017–0.026; *wInd* = 0.014–0.019), Con (*wSit* = 0.020–0.026; *wCon* = 0.022–0.027; *wGel* = 0.014–0.019; *wStri* = 0.019–0.024) and Per *(wPer* = 0.014–0.020) (Table 2). In contrast, high values of genetic distances (0.234–0.255) were found by comparing the strains clustered into the Supergroup A when *wNiv* from *Aedes* (*Stegomyia*) *niveus* was included (Table 2).

The haplotype *wLcy* showed low genetic distance values in comparison to strains located in Subgroup B, such as Prn (*wPrn* = 0.017) (Table 2) identified in the phlebotomine *Phlebotomus perniciosus*; strains *wInd*, *wUnif* (Group Unif = 0.019) out of group Unif; strains *wSit* (0.024), *wCon* (0.027) *wGel* (0.019), *wStri* (0.019) out of group Con, detected in mosquito species *Mansonia indiana*, *Mansonia uniformis* and *Culex gelidus*; and in the homopteran *Laodelphax striatellus*, respectively (Table 2, Additional file 1). Both *wLev* and w*Lcy* showed higher values of genetic distances in relation to *Wolbachia* strains in Supergroup A, among which *wNiv* (0.240), *wPa* (0.230) and *wSub* (0.231) are highlighted.

The percentage divergence based on alignment, which includes a large number of available sequences, suggests that *wsp* gene sequences from *Wolbachia* present considerable intra- and inter-genic variation. This can be summarized as follows: between sequences of the same strain there is 0.4% variation; between strains of the same group there is 1–2.1% variation; between strains of different groups located in the same supergroup there is 1.9–2.7% variation; and between strains of different supergroups there is 13.4–25.5% variation (Table 2). These percentages are consistent with the established ranges for the separation of strains and current assignment of *Wolbachia* groups [27].

Phylogenetic relationships estimated by Bayesian Inference analysis (including 449 bp in the final alignment) grouped the strains *wLev* and *wLcy* in a new group called "*wLeva*" (branch support of 0.97), located in the Supergroup B, and based on the robustness of clade posterior probability (0.71) with respect to Supergroup A (Fig. 3). The *Leva* group has a close phylogenetic relationship (0.98) with the Dei, Crag, Unif, and Prn groups (Fig. 3).

### *Leishmania* infection

Eighteen female groups composed of 171 individual specimens of *Lu. evansi*, *Lu. dubitans*, *Lu. c. cayennensis*, *Lu. gomezi*, *Lu. trinidadensis*, *Lu. rangeliana* and *Lu. atroclavata*, were negative for *Leishmania* infection.

## Discussion

This study reports a natural infection of endosymbiotic *Wolbachia* in natural populations of *Lu. dubitans*, *Lu. c. cayennensis* and *Lu. evansi* for the first time from the peri-urban environment of a leishmaniasis focus transmission on the Caribbean coast of Colombia.

Different studies with similar sample sizes (between 141 and 547 individuals) and grouping of individuals by species (10–100) have been developed, and determine infection rates [32]. We decided not to do calculations infection rates from DNA *Lutzomyia* groups because we consider that the prevalence of *Wolbachia* may be low and poorly estimated. For this reason, we only emphasize on infected species and characterization of genetic haplotypes.

*Lutzomyia evansi* and *Lu. dubitans* were found to be infected with *Wolbachia* by a strain named *wLev*, while *Lu. c. cayennensis* was infected with both strains of *Wolbachia* (*wLcy* and *wLev*). This is consistent with the presence of these insect species in a uniform ecological region (similar collection localities). Regarding *Lu. c. cayennensis*, there exists a possibility that *Wolbachia* infected this species more than once, which would explain the presence of two different strains. In some studies, some *Wolbachia* strains belonging to different subgroups or groups have been observed to infect the same host species [33].

The groupings based on *Wolbachia wsp* gene sequences included in this study were well supported and consistent with those previously reported for Supergroups A and B [34]. The *Wolbachia* strains *wLev* and *wLcy* reported in this study appear to be included as a group in Supergroup B, which is common in arthropods. *Wolbachia* strains *wLev* and *wLcy* show close relationships to the Prn, Con and Unif groups of Supergroup B [12]. Proximity to the group Prn is highlighted, because the *wPrn* strain was found in the host *Ph. pernisiosus* [12]. In contrast, strains *wLcy* and *wLev* located in this group do not appear to show a close relationship to *Wolbachia* strains in group *Whi* (*Lu. whitmani* and *Lu. shannoni*), which are detected in species of the subfamily Phlebotominae, even though they have a closely related host and a similar continental distribution [23]. Interestingly, some strains of Supergroup B (*wPip*, *WBoL* and *wVul*) have phenotypes associated with feminization of males, as well as mortality and cytoplasmic incompatibility [35]. Each of these reproductive alterations are advantageous to *Wolbachia* as they are correlated to an increase in infected females. This group of strategies is called reproductive parasitism [36].

The species *Lu. evansi*, *Lu. dubitans* and *Lu. c. cayennensis* were found positive for *Wolbachia* infection both by PCR and by sequencing of the *wsp* gene, that enables a fast assigning of unknown strains to a particular group [37]. These three species have a history of natural infection by species of *Leishmania* [1, 3]. However, in this study, *Leishmania* was not detected in them. The prevalence of natural infections with *Leishmania* in sand flies is low. The process of simultaneous identification of *Leishmania* and *Wolbachia* can be complicated and needs to be initially standardized under laboratory conditions. Other researchers have reported differences in the sensitivity of different molecular markers and conventional tests (PCR, RFLP, isozyme patterns, hybridization with DNA probes) for the detection, diagnosis and identification of *Leishmania* species [37]; and they propose that exploring the possibility of viewing promastigotes by the dissection of digestive tracts and the implementation of more variants of PCR with genus-specific primers would be beneficial. Also it is necessary to indicate that the absence of *Wolbachia* and *Leishmania* in *Lutzomyia* species may be influenced by the sampling scheme (spot scouting) and the size of the analyzed sample, which reduces the possibility of detecting positive DNA of *Leishmania*. Identification of species of *Leishmania* from vectors has also been constrained by the need to isolate the parasite from one or more of the small proportion of sand flies that are normally found to be infected, ranging from 0.001 to 2.26% for *Leishmania* transmission [37].

It is desirable to advance our understanding of the biology and spread of *Wolbachia* bacteria in relation to *Leishmania* infection, given the fact that different studies show the impact of these bacteria in host-parasite interactions with a potential use in reducing the risk of infectious diseases caused by parasites and transmitted to humans by insects [38]. Many invertebrates are infected by *Wolbachia*, and the bacteria’s success may be credited to the diverse phenotypes (mutualism or reductive parasitism) that result from infection. The persistence of the *Wolbachia* infections and phenotype estimation in natural populations of *Lutzomyia* in the municipality of Ovejas, are determinants to make strong correlations of the role of *Wolbachia* on the development of *Leishmania*. Another area of study, may include the introduction of *Wolbachia* in *Lutzomyia evansi* (main vector and abundant species in the Caribbean coast) and its interaction with *Leishmania*.

Additionally, it has been found that the presence of some strains of *Wolbachia* in mosquitoes can regulate the expression of genes involved in the immune responses, resulting in inhibition of the replication, multiplication, or resistance to the proliferation of viruses, parasites, and microfilariae [39]. In this sense, *Wolbachia* can also be visualized as a microorganism for biological control, that is based on the substitution of the microbiome of the vector by microorganisms that affect vector’s pathogen load. Replacement microbiota may represent unmodified microbial species that normally do not colonize a particular vector species, or genetically engineered symbiotic bacteria [40]. A vector’s microbiome can be altered either through the stable “conversion” of vector populations in the wild or by introducing the desirable microbiota through bait stations [40, 41], which allows for a continuous modification of vector populations.

## Conclusions

Our study represents a significant advance in the understanding of natural infections of *Wolbachia* in *Lutzomyia*. Further studies are needed to investigate the dynamics of infections with *Wolbachia* and *Leishmania* in natural populations of *Lutzomyia* present in other areas of leishmaniasis transmission.

## Additional file

Additional file 1: Nomenclature of *Wolbachia* supergroups, groups, strains used and host insects related. These strains were used to compare genetic distances and perform phylogenetic reconstructions. (DOCX 20 kb)

## Abbreviations

*cox*1: Cytochrome *c* oxidase subunit 1
*Le*: *Leishmania*
*Lu*: *Lutzomyia*
*wsp*: Gene coding for the main surface protein of endosymbiotic *Wolbachia*

## References

[1] Cortés A, Pérez-Doria A, Bejarano E (2009). Flebotomíneos (Diptera: Psychodidae) antropofílicos de importancia en salud pública en Los Montes de María. Rev Cuba Med Trop.

[2] Bates PA, Depaquit J, Galati EAB, Kamhawi S, Maroli M, Mcdowell MA, et al. Recent advances in phlebotomine sand fly research related to leishmaniasis control. Parasit Vectors. 2015;8:131. 10.1186/s13071-015-0712-x PMC4352286 25885217

[3] Cochero BS, Anaya EY, Díaz EY, Paternina EM, Luna EA, Luis E. Infección natural de *Lutzomyia cayennensis cayennensis* con parásitos tripanosomatídeos (Kinetoplastida: Trypanosomatidae) en Los Montes de María, Colombia. Rev Cuba Med Trop. 2007;59:35–9. 23427416

[4] Sharma U, Singh S (2008). Insect vectors of *Leishmania*: distribution, physiology and their control. J Vector Borne Dis.

[5] Jeffries CL, Walker T (2016). *Wolbachia* biocontrol strategies for arboviral diseases and the potential influence of resident *Wolbachia* strains in mosquitoes. Curr Trop Med Reports.

[6] Serbus LR, Casper-Lindley C, Landmann F, Sullivan W (2008). The genetics and cell biology of *Wolbachi*a-host interactions. Annu Rev Genet.

[7] Rossi P, Ricci I, Cappelli A, Damiani C, Ulissi U, Mancini MV (2015). Mutual exclusion of *Asaia* and *Wolbachia* in the reproductive organs of mosquito vectors. Parasit Vectors.

[8] Blagrove MS, Arias-Goeta C, Failloux AB, Sinkins SP. *Wolbachia* strain *w*Mel induces cytoplasmic incompatibility and blocks dengue transmission in *Aedes albopictus*. Proc Natl Acad Sci USA. 2012;109:255–60. 10.1073/pnas.1112021108 PMC3252941 22123944

[9] Moreira LA, Iturbe-Ormaetxe I, Jeffery JA, Lu G, Pyke AT, Hedges LM (2009). A *Wolbachia* symbiont in *Aedes aegypti* limits infection with dengue, chikungunya and *Plasmodium*. Cell.

[10] Hughes GL, Koga R, Xue P, Fukatsu T, Rasgon JL (2011). *Wolbachia* infections are virulent and inhibit the human malaria parasite *Plasmodium falciparum* in *Anopheles gambiae*. PLoS Pathog.

[11] Matsumoto K, Izri A, Dumon H, Raoult D, Parola P (2008). First detection of *Wolbachia* spp., including a new genotype, in sand flies collected in Marseille, France. J Med Entomol.

[12] Ono M, Braig HR, Munstermann LE, Ferro C, O’Neill SL (2001). *Wolbachia* infections of phlebotomine sand flies (Diptera: Psychodidae). J Med Entomol.

[13] Azpurua J, De La Cruz D, Valderama A, Windsor D. *Lutzomyia* sand fly diversity and rates of infection by *Wolbachia* and an exotic *Leishmania* species on Barro Colorado Island, Panama. PLoS Negl Trop Dis. 2010;9(4):3. 10.1371/journal.pntd.0000627 PMC2834748 20231892

[14] Hughes GL, Rasgon JL (2014). Transinfection: A method to investigate *Wolbachia*-host interactions and control arthropod-borne disease. Insect Mol Biol.

[15] Brownstein JS, Hett E, O’Neill SL (2003). The potential of virulent *Wolbachia* to modulate disease transmission by insects. J Invertebr Pathol.

[16] Parvizi P, Bordbar A, Najafzadeh N. Detection of *Wolbachia pipientis*, including a new strain containing the *wsp* gene, in two sister species of *Paraphlebotomus* sand flies, potential vectors of zoonotic cutaneous leishmaniasis. Mem Inst Oswaldo Cruz. 2013;108:1–7. 10.1590/0074-0276108042013004 PMC3970627 23828002

[17] Mirkery-Pachecho O, Marina C, Ibañez B, Sanchez D, Castillo V. Infeccion natural de *Lutzomyia cruciata* (Diptera: Psychodidae, Phlebotominae) con *Wolbachia* en cafetales de Chiapas, México. Act Zoológica Mex. 2012;8(2):401–13.

[18] Island C, Azpurua J, Cruz DD L, Valderama A, Windsor D (2010). *Lutzomyia* sand fly diversity and rates of infection by *Wolbachia* and an exotic *Leishmania* species on Barro.

[19] Bordbar A, Soleimani S, Fardid F, Zolfaghari MR, Parvizi P (2014). Three strains of *Wolbachia* pipientis and high rates of infection in Iranian sandfly species. Bull Entomol Res.

[20] Parvizi P, Benlarbi M, Ready PD (2003). Mitochondrial and *Wolbachia* markers for the sandfly *Phlebotomus papatasi*: Little population differentiation between peridomestic sites and gerbil burrows in Isfahan province. Iran Med Vet Entomol.

[21] Kassem HA, Osman G (2007). Maternal transmission of *Wolbachia* in *Phlebotomus papatasi* (Scopoli). Ann Trop Med Parasitol.

[22] Baldo L, Hotopp JCD, Jolley KA, Bordenstein SR, Biber SA, Choudhury RR (2006). Multilocus sequence typing system for the endosymbiont *Wolbachia* pipientis. Appl Environ Microbiol.

[23] Young DG, Duncan M. Guide to the identification and geographic distribution of *Lutzomyia* sand flies in Mexico, the West Indies, Central and South America. J Biol Sci. 1994.

[24] Golczer G, Arrivillaga J (2008). Modificación de un protocolo estándar de extracción de ADN para flebotominos pequeños (Phlebotominae: *Lutzomyia*). Rev Colomb Entomol.

[25] Braig HR, Zhou W, Dobson SL, O’Neill SL (1998). Cloning and characterization of a gene encoding the major surface protein of the bacterial endosymbiont *Wolbachia pipientis*. J Bacteriol.

[26] Werren JH, Zhang W, Guo LR (1995). Evolution and phylogeny of *Wolbachia*: reproductive parasites of arthropods. Proc R Soc B.

[27] Zhou W, Rousset F, O’Neil S (1998). Phylogeny and PCR-based classification of *Wolbachia* strains using wsp gene sequences. Proc Biol Sci.

[28] Hall T (1999). BioEdit: a user-friendly biological sequence alignment editor and analysis program for Windows 95/98/NT. Nucleic Acids Symp Ser.

[29] Darriba D, Taboada G, Doallo R, Posada D (2012). jModelTest 2: more models, new heuristics and parallel computing. Nat Methods.

[30] Guindon S, Dufayard J, Lefort V, Anisimova M, Hordijk W, Gascuel O (2010). New algorithms and methods to estimate maximum-likelihood phylogenies: assessing the performance of PhyML 3.0. Syst Biol.

[31] Fraga J, Montalvo AM, De Doncker S, Dujardin J-C, Van der Auwera G (2010). Phylogeny of *Leishmania* species based on the heat-shock protein 70 gene. Infect Genet Evol.

[32] Marcon HS, Coscrato VE, Selivon D, Perondini AL, Marino CL (2011). Variations in the sensitivity of different primers for detecting *Wolbachia* in *Anastrepha* (Diptera: Tephritidae). Brazilian J Microbiol.

[33] Kondo N, Shimada M, Fukatsu T (2005). Infection density of *Wolbachia* endosymbiont affected by co-infection and host genotype. Biol Lett.

[34] Hinrich J, Schulenburg GVD, Hurst GDD, Huigens TME, Van Meer MMM, Jiggins FM (2000). Molecular evolution and phylogenetic utility of *Wolbachia* ftsZ and wsp gene sequences with special reference to the origin of male-killing. Mol Biol Evol.

[35] Jiggins FM, Bentley JK, Majerus ME, Hurst GD (2001). How many species are infected with *Wolbachia*? Cryptic sex ratio distorters revealed to be common by intensive sampling. Proc Biol Sci.

[36] Zug R, Hammerstein P. Bad guys turned nice? A critical assessment of *Wolbachia* mutualisms in arthropod hosts. Biol Rev. 2014 10.1111/brv.12098 24618033

[37] Pintureau B, Chaudier S, Lassabliere F, Charles H, Grenier S (2000). Addition of *wsp* sequences to the *Wolbachia* phylogenetic tree and stability of the classification. J Mol Evol.

[38] Slatko BE, Luck AN, Dobson SL, Foster JM. *Wolbachia* endosymbionts and human disease control. Mol Biochem Parasitol. 2014; 88–95 10.1016/j.molbiopara.2014.07.004 25046729

[39] LePage D, Bordenstein SR (2013). *Wolbachia*: Can we save lives with a great pandemic?. Trends Parasitol.

[40] Leitner WW, Wali T, Kincaid R, Costero-Saint DA (2015). Arthropod vectors and disease transmission: translational aspects. PLoS Negl Trop Dis.

[41] Walker T, Johnson PH, Moreira LA, Frentiu FD, Mcmeniman CJ, Leong YS (2011). The *wMel Wolbachia* strain blocks dengue and invades caged *Aedes aegypti* populations. Nature.

