# Supplementary material for: Molecular detection and identification of Wolbachia in three species of the genus Lutzomyia on the Colombian Caribbean coast
Source: Parasit Vectors. 2017 Feb 28;10:110. doi: 10.1186/s13071-017-2031-x (PMC5329942; doi:10.1186/s13071-017-2031-x)
Supplement: Additional file 1: — Nomenclature of Wolbachia supergroups, groups, strains used and host insects related. These strains were used to compare genetic distances and perform phylogenetic reconstructions. (DOCX 20 kb) [file 13071_2017_2031_MOESM1_ESM.docx]

**Additional file 1. Nomenclature of *Wolbachia* supergroups, groups, strains used and host insects related.** These strains were used to compare genetic distances and perform phylogenetic reconstructions.

| **Supergroups** | **Group** | | **Host insects (strain of *Wolbachia*)** | **Accession number (GenBank)** | **References** |
| --- | --- | --- | --- | --- | --- |
| A | Mel | *Drosophila melanogaster (Wmel* positive control, transfected in [*Aedes* (*Stegomyia aegypti*](http://es.wikipedia.org/wiki/Aedes_aegypti)*), wMelCS, wMelH), D. simulans (wCof)* | | AF020072-AF020063- AF020065 -AF020064-AF020066-AF020067 | Zhou et al., 1998 |
|  | AlbA | *Aedes albopictus (wAlbA)* | | AF020059 | Zhou et al., 1998 |
|  | Mors | *Glossina morsitans (wMors), Nasonia vitripennis (wVitA), Glossina centralis (wCen), Ae. albotaeniatus (wAlbo), Tripterioides aranoides (wAra), Cx. brevipalpis (wBre), Hodgesia* spp. (*wHod*) | | AF020079 - AF020081-AF020078- AF317475- AF317476- AF317477- AF317483 | Zhou et al., 1998, Ono et al., 2001, Ruang-Areerate et al., 2003 |
|  | Riv | *D. simulans (Riverside) y D. auraria (wRi)* | | AF020070 - AF020062 | Zhou et al., 1998 |
|  | Uni | *Muscidifurax uniraptor (wUni)* | | AF020071 | Zhou et al., 1998 |
|  | Haw | *D. simulans (Hawaii), D. sechellia (wHa), Cadra cautella* (*wCauA*) | | AF020068- AF020073- AF020075 | Zhou et al., 1998 |
|  | Pap | *Phlebotomus papatasi*, *P. mongolensis (wPap, Turk54), P. caucasicus (Turk54), P. perfiliewi transcaucasicus, P. kandelakii. (wPap)* | | AF020082 - AF237882 - AF237883- EU780683- KC576916 | Zhou et al., 1998, Ono et al., 2001, Parvizi et al., 2013, Parvizi et al., 2012 |
|  | Aus | *Glossina austeni* (*wAus*) | | AF020077 | Zhou et al., 1998 |
|  | Whi | *Lutzomyia whitmani, Lutzomyia shannoni* (*wWhi*) | | AF237885- AF237886 | Ono et al., 2001 |
|  | Lop | *Culex* (*Lophoceraomyia*) spp. | | AF317490 | Ruang-Areerate et al., 2003 |
|  | Eum | *Cx. (Eumelanomyia)* spp*. ( wEum)* | | AF317480 | Ruang-Areerate et al., 2003 |
|  | Nov | *Ae. novoniveus ( wNov )* | | AF317484 | Ruang-Areerate et al., 2003 |
|  | Niv | *Ae. niveus (wNiv)* | | AF317485 | Ruang-Areerate et al., 2003 |
|  | Sub | *Armigeres subalbatus (wSub)* | | AF317488 | Ruang-Areerate et al., 2003 |
| **B** | Con | *Tribolium confusum (wCon), Cx. Gelidus (wGel), Laodelphax striatellus (wStri), Cx. Sitiens (wSit)* | | AF020083- AF317482 - AF020080 - AF317491 | Zhou et al., 1998, Ruang-Areerate et al., 2003 |
|  | Dei | *Trichogramma deion (wDei)* | | AF020084 | Zhou et al., 1998 |
|  | Pip | *Culex quinquefasciatus, Culex pipiens (wPip), Cx. Fuscocephala (wFus), Drosophila simulans (mauritiana)* y *Ephestia Kuehniella (wMa), Drosophila simulans (Noumea) (wNo), Aedes albopictus (wAlbB), Ae. pseudalbopictus ( wPseu), Ar. Kesseli (wKes)* | | AF020061, AF020060, AF317481-AF020069 -AB024570-AF020074-AF020059- AF317487- AF317489 | Zhou et al., 1998, Ono et al., 2001, Ruang-Areerate et al., 2003 |
|  | CauB | *Ephestia cautella (wCauB), Tagosodes orizicolus (wOri)* | | AF020076 - AF020085 | Zhou et al., 1998, |
|  | Prn | *Phlebotomus perniciosus (wPrn)* | | AF237884 | Ono et al., 2001, |
|  | Dro | *Trichopria drosophilae, Asobara tabida (WDro)* | | AF071910- AF124856 | Zhou et al., 1998, |
|  | Unif | *Mansonia indiana (*wInd*), Mn. Uniformis (wUnif*) | | AF317492- AF317493 | Ruang-Areerate et al., 2003 |
|  | Crag | *Ae. Craggi (WCrag*) | | AF317478, AF317479 | Ruang-Areerate et al., 2003 |
|  | Perp | *Ae. perplexus (wPerp)* | | AF317486 | Ruang-Areerate et al., 2003 |
|  | **Leva** | ***Lu. evansi* (*wLev*)*, Lu. dubitans* (*wLev*)*, Lu.cayennensis* (*wLeva-wLcay*)** | | KR907869, KR907870, KR907871, KR907872, KR907873, KR907874 | --- |
| **Outgroup** | | *Bemicia tabaci* | | FJ404651, FJ404653 | Parvizi et al., 2013 |
